# The relationship between 11 different polygenic longevity scores, parental lifespan, and disease diagnosis in the UK Biobank

**DOI:** 10.1007/s11357-024-01107-1

**Published:** 2024-03-07

**Authors:** Janith Don, Andrew J. Schork, Gwênlyn Glusman, Noa Rappaport, Steve R. Cummings, David Duggan, Anish Raju, Kajsa-Lotta Georgii Hellberg, Sophia Gunn, Stefano Monti, Thomas Perls, Jodi Lapidus, Laura H. Goetz, Paola Sebastiani, Nicholas J. Schork

**Affiliations:** 1 Translational Genomics Research Institute (TGen), Phoenix, AZ USA; 2 The Institute of Biological Psychiatry, Copenhagen University Hospital, Copenhagen, Denmark; 3 GLOBE Institute, Copenhagen University, Copenhagen, Denmark; 4 Institute for Systems Biology, Seattle, WA USA; 5 San Francisco Coordinating Center, California Pacific Medical Center Research Institute, San Francisco, CA USA; 6 Department of Biostatistics, Boston University School of Public Health, Boston, MA USA; 7 Department of Biostatistics, Oregon Health & Science University, Portland, OR USA; 8 Veterans Affairs Loma Linda Health Care, Loma Linda, CA USA; 9 Department of Biostatistics, Boston University School of Public Health, Boston, MA USA; 10 Institute for Clinical Research and Health Policy Studies, Tufts Medical Center, Boston, MA USA; 11 Tufts University School of Medicine and Data Intensive Study Center, Boston, MA USA; 12 The City of Hope National Medical Center, Duarte, CA USA; 13 Department of Medicine, Section of Geriatrics, Boston University, Boston, MA USA

**Keywords:** Polygenic risk score, Polygenic longevity score, Lifespan, Association analysis, Disease risk, Variant annotations

## Abstract

Large-scale genome-wide association studies (GWAS) strongly suggest that most traits and diseases have a polygenic component. This observation has motivated the development of disease-specific “polygenic scores (PGS)” that are weighted sums of the effects of disease-associated variants identified from GWAS that correlate with an individual’s likelihood of expressing a specific phenotype. Although most GWAS have been pursued on disease traits, leading to the creation of refined “Polygenic Risk Scores” (PRS) that quantify risk to diseases, many GWAS have also been pursued on extreme human longevity, general fitness, health span, and other health-positive traits. These GWAS have discovered many genetic variants seemingly protective from disease and are often different from disease-associated variants (i.e., they are not just alternative alleles at disease-associated loci) and suggest that many health-positive traits also have a polygenic basis. This observation has led to an interest in “polygenic longevity scores (PLS)” that quantify the “risk” or genetic predisposition of an individual towards health. We derived 11 different PLS from 4 different available GWAS on lifespan and then investigated the properties of these PLS using data from the UK Biobank (UKB). Tests of association between the PLS and population structure, parental lifespan, and several cancerous and non-cancerous diseases, including death from COVID-19, were performed. Based on the results of our analyses, we argue that PLS are made up of variants not only robustly associated with parental lifespan, but that also contribute to the genetic architecture of disease susceptibility, morbidity, and mortality.

**Supplementary Information:**

The online version contains supplementary material available at 10.1007/s11357-024-01107-1.

## Introduction

While rare genetic variants are primary causal factors for several infrequent yet debilitating diseases, common chronic diseases (e.g., cardiovascular disease, Alzheimer’s disease, and diabetes) typically have a multifactorial and polygenic basis influenced by the cumulative impact of thousands of common genetic variants. In fact, genome-wide association studies (GWAS) have identified over 200,000 genetic variants associated with a wide variety of traits and diseases [1]. The allelic effects of most, but not all, of these variants are small. It is now widely accepted that a large fraction of human phenotypic variation has a polygenic basis such that small effects of individual variants can have a cumulative effect on phenotypes that is pronounced [2–4]. A complete understanding of how a polygenic background influences phenotypic variation against highly variable environments in the population at large is currently lacking, but is of considerable interest to evolutionary biologists, epidemiologists, and clinical researchers for obvious reasons [3].

The recognition of a polygenic basis for most phenotypes has motivated the development of “polygenic scores” (PGS) which are weighted sums of the effects of potentially thousands of variants in individuals’ genomes based on estimates from GWAS that capture or index aspects of individuals’ underlying genetic predisposition to express a particular phenotype [4–7]. “Polygenic Risk Scores (PRS),” which are essentially PGS applied to different diseases, have been shown to identify individuals at risk of specific diseases as reliably as traditional single locus–based genetic tests for many rare monogenic conditions [4, 5, 7]. In addition, it has been shown that the high penetrance of some rare disease-causing variants (e.g., BRCA variants and breast cancer) can be modified substantially by PRS [8–10]. This suggests that PRS have important clinical utility. However, there are many well-recognized impediments to the routine use of PRS in clinical settings, including a need to better understand their interactions with environmental factors, how they can complement measures of current health state (e.g., blood pressure or cholesterol level), whether they are modified by genetic ancestry, and whether they run afoul of current ethical, legal, and social norms assumed in routine primary care [4–7, 11–16].

The development and construction of PGS/PRS have been the focus of many methodological studies, and these studies have provided effective tools for constructing reliable PGS/PRS [15, 17–19]. These tools allow PGS/PRS to be derived from very large data sets or meta-analyses [20–22], and open-source websites have been developed that provide the information needed to compute PRS for over 3200 traits and diseases [13, 23]. The ubiquity of PGS/PRS methods and the availability of large data sets have motivated studies of the polygenic basis of many non-disease traits, such as height [24], as well as health-positive traits such as health span [25], beneficial disease treatment response [26–28], and resilience to disease and longevity [29–34]. PGS computed for health-positive traits has raised many important questions. For example, it is of interest to know whether variants associated with longevity simply reflect the alternative alleles at loci harboring disease-associated variants; whether the variants associated with longevity overlap with variants associated with other phenotypes, including disease phenotypes [35, 36]; and whether variants associated with longevity appear to be protective against the development of diseases generally, or protective of only a few specific diseases.

Some of these questions have been recently explored by Tesi et al. [33] and Torres et al. [37] who developed polygenic models predictive of longevity—what we refer to as “Polygenic Longevity Scores” or PLS—based on GWAS data. Unfortunately, as noted in the studies by Tesi et al. [33] and Torres et al. [37], identifying variants associated with longevity is complicated. Different definitions of longevity are used in different studies, sample sizes in studies focusing on extreme longevity (EL) are small due to the rarity of extremely old (and healthy) individuals, and it can be difficult to account for population stratification, environmental exposures, dietary practices, and behaviors in relevant studies [32, 38, 39]. However, concerted efforts to pursue GWAS on EL beyond those pursued by Tesi et al. [33] and Torres et al. [37] have led to meta-analyses of many different GWAS of EL [29, 32]. In addition, GWAS focusing on parental lifespan as a surrogate for individual lifespan have been pursued using more than 1 million individuals, including individuals in the UK Biobank (UKB) [34]. Interestingly, many of these studies suggest that long-lived individuals exhibit differences in disease PRS profiles as compared short-lived individuals, raising questions about the relationships between PLS, PRS, longevity, and disease [29, 40–42].

We explored the relationships between 11 different PLS derived from 4 GWAS of longevity and parental lifespan, disease diagnosis, and population structure in the UKB. We used GWAS summary statistics from Deelen et al. [29], Timmers et al. [34], Sebastiani et al. [32], and Tesi et al. [33]. We note that since the GWAS by Timmers et al. [34] and Tesi et al. [33] were pursued, in part, with data from the UKB, there is a level of training bias when exploring them with other UKB data. We highlight this in our analyses by distinguishing them from the other PLS. The PLS we considered used different, yet intuitive, criteria for their derivation, allowing us to contrast their derivation and association strength with parental lifespan and disease diagnoses; for example, the use of different criteria and data sets for the derivation of the 11 PLS allowed us to explore differences in the single nucleotide polymorphisms (SNPs) and the effect sizes assigned to each.

## Methods

### The construction of 11 different PLS

We obtained and downloaded the summary statistics from the GWAS pursued by Deelen et al. [29], Timmers et al. [34], Sebastiani et al. [32], and Tesi et al. [33] from material in their publications, public repositories, or permission from the research teams that conducted the studies. We used simple variant weighting schemes to construct PLS based on the effect size of each associated variant from the different GWAS. We ultimately used different criteria to create 11 PLS from the 4 GWAS (see Table 1). For the GWAS by Deelen et al. [29], denoted “dl” in the names we associated with each PLS (Table 1), we constructed a PLS based on the SNPs reported as significantly associated with longevity among participants with European ancestry (Table 2 of [29]) where the definition of long-lived individuals included those with ages greater than the 90th (dl90eur) and 99th (dl99eur) percentiles of relevant age and sex-specific survival distributions. Furthermore, we also used a *p* value threshold of *p* < 5e-8 on dl90eur and dl99eur to filter in the other significant variants and create another two PLS: dl90eur5_8e and dl99eur5_8e, respectively. We also computed broader PLS implicating a large number of variants using the “PRS-CS (polygenic prediction via continuous shrinkage priors)” software [43] from the summary statistics of the GWAS on subjects > 90th (dl90_cs) and 99th (dl99_cs) percentiles of the survival distributions. For the GWAS by Timmers et al. [34], denoted “tim,” we constructed a PLS based on all reported significant variants (tim) as well as the application of the PRS-CS program to the GWAS summary statistics (tim_cs) by Timmers et al. [34]. For the GWAS by Sebastiani et al. [32], denoted “seb,” we constructed a PLS from all reported significant variants for individuals with age > 99th survival percentile (seb), as well as the application of the PRS-CS program (seb_cs). For the study by Tesi et al. [33], we used their reported best performing set of variants (tesi) and their respective effect size values.

**Table 1:** Summary of the PLS used in the current study

PLS label	Criteria	Reference	No. of variants	No. of variants used with UKB^a^
dl90eur	All reported significant SNPs for age > 90th survival percentile reported in Table 2 of Deelen et al	[29]	7	7
dl90eur5_8e	SNPs in dl90eur with a *p* value < 5E-8		3	3
dl99eur	All reported significant SNPs for age > 99th survival percentile reported in Table 2 of Deelen et al		6	3
dl99eur5_8e	SNPs in dl99eur with a *p* value < 5E-8		2	2
dl90_cs	PRS-CS applied to age > 99th survival percentile summary statistics		2,659,680	1,108,009
dl99_cs	PRS-CS applied to age > 99th survival percentile summary statistics		2,645,188	1,105,968
tim	Significant SNPs reported in the paper	[34]	19	18
tim_cs	PRS-CS applied to summary statistics		9,085,648	1,100,079
seb	All reported significant SNPs for age > 99th survival percentile	[32]	10	10
seb_cs	PRS-CS applied to summary statistics		6,208,151	977,820
tesi	Reported best performing set of SNPs in re-analyses of Timmers et al.’s GWAS	[34]	94	94

^a^This column consists of the number of variants finally used to calculate PLS in UK Biobank individuals after all the filtering

**Table 2:** Number of variants in the UKB (along the diagonal, in italics), those in common among the different PLS (above the diagonal), and pairwise metric of variant weight overlap (below the diagonal)

PLS	dl90eur	dl90eur5_8e	dl99eur	dl99eur5_8e	dl90_cs	dl99_cs	tim	tim_cs	seb	seb_cs	tesi
dl90eur	*7*	3	2	2	1	1	1	2	0	2	2
dl90eur5_8e	0.225	*3*	2	2	0	0	1	1	0	1	2
dl99eur	0.186	0.297	*3*	2	0	0	1	1	0	1	2
dl99eur5_8e	0.224	0.357	0.445	*2*	0	0	1	1	0	1	2
dl90_cs	0.000	0.000	0.000	0.000	*1,108,009*	1,105,743	1	1,090,918	2	973,879	23
dl99_cs	0.000	0.000	0.000	0.000	0.000	*1,105,968*	1	1,088,901	2	973,389	23
tim	0.043	0.069	0.082	0.098	0.000	0.000	*18*	1	0	1	10
tim_cs	0.000	0.000	0.000	0.000	0.000	0.000	0.000	*1,100,079*	2	962,783	23
seb	0.000	0.000	0.000	0.000	0.000	0.000	0.000	0.000	*10*	2	0
seb_cs	0.000	0.000	0.000	0.000	0.000	0.000	0.000	0.000	0.000	*977,820*	19
tesi	0.036	0.058	0.071	0.086	0.000	0.000	0.021	0.000	0.000	0.000	*94*

We emphasize that there are multiple approaches for constructing PGS, and ultimately PLS. The most basic method is to use only significant variants (using some *p* value threshold) that are not in linkage disequilibrium (LD) with other variants. Using only independent variants mitigates the effect of “double counting” the weights or number of variants used in the PRS/PLS calculations. Recently, a number of approaches, such as the PRS-CS approach we used, have been shown to improve the power of PGS by adjusting effect sizes for all variants across the genome using LD information and various association strength significance levels [20, 43]. In addition, we also only considered individuals of British Caucasian ancestry in the UKB in our analyses to reduce the effects of heterogeneity. The “–score” function in the PLINK 2.0 software was used to construct the polygenic scores from the selected individual genotypes and allelic effects of selected variants [44].

### Application of the PLS to the UKB genotype data

The UKB cohort contains genotyped data for around 480,000 individuals from the UK aged between 40 and 69 at the time of recruitment during the years 2006–2010 [45]. We used the intersection of ~ 30 million high-quality imputed variants (information score > 0.8 from ~ 96 million variants from imputed data version 3) from the UKB and variants selected for our 11 proposed PLS (Table 1). We note that some of the models using PRS_CS to compute PLS used only variants passing quality filters implemented in the PRS_CS package (HapMap3 SNPs with imputation information score > 0.8 and minor allele frequency > 1%). The last column of Table 1 shows the number of variants used in the final PLS calculations. It is important to emphasize that not all SNPs from the four original GWAS publications and our construction of PLS based on them were actually genotyped or imputed reliably in the UKB. This led to discrepancies between the computation of the PLS from the initial sets of SNPs and the computation with the UKB data, with some PLS being based on fewer SNPs in the UKB than in the original publications. This likely leads to a more conservative set of PLS as studied in the UKB. We note that we did this to avoid having to impute or assign weights to be used in the revised scoring and other issues (e.g., frequency differences among different potential proxy variants).

### PRS construction

We computed disease PRS for the UKB participants for the following non-cancer diseases: Alzheimer’s disease, atrial fibrillation, coronary artery disease, coronary heart disease, celiac disease, type 1 diabetes, and type 2 diabetes, using information from available PRS catalogs and databases [23, 46–53]. These PRS were used in comparisons and contrasts with PLS associations with specific disease associations. For cancers, we computed PRS for bladder, breast, colorectal, leukemia, lung, oropharyngeal, ovarian, pancreatic, prostate, testicular, and thyroid, using allelic effects from various published GWAS sources and database [23, 54, 55]. We chose these PRS since the diseases they were developed for are known to be age-related.

### PLS association analyses

As noted, we confined attention to British individuals of European ancestry to reduce confounding effects from both ancestry and gross differential environmental effects individuals of different ancestries are exposed to. We extracted this group by selecting “White British” from self-reported ancestral background (UKB data field 21,000). From the extracted group, we selected “Caucasians” from genetic ethnic group information in the UKB (UKB data field 22,006, which is the largest single ethnic group at > 400,000 individuals characterized by UKB genetic data).

To explore commonalities among the variants used to construct the different PLS, we first tallied the number of variants common to each pair of PLS. We further defined a new metric of “variant weight overlap” to compare each pair of PLS in terms of the weight contribution of their shared variants. Denoting the weight of variant a in PLS *x* as *w*_a,*x*_, we compute the total weight of variants in PLS *x* as ${T}_{x}={\sum }_{i}^{x}\left|{w}_{i,x}\right|$, and the variant weight overlap (VWO) between PLS *x* and PLS *y* as ${{\text{VWO}}}_{x,y}={\sum }_{i}^{x\cap y}\left|\frac{{w}_{i,x}{w}_{i,y}}{{T}_{x}{T}_{y}}\right|$, where $x\cap y$ denotes the set of variants shared by both PLS lists. Thus, two identical lists would yield a value of 1, two disjoint lists would yield a value of 0, and in the case of two pairs of lists with an equal number of shared variants, the resulting value will be higher when the shared variants contribute larger weight to each PLS. We also computed the Spearman and Pearson correlations among PLS values after they were computed on the UKB participants, where the differences in the number of variants used in each is likely to have an impact on these correlations.

We tested the association of each of the 11 PLS with parental lifespan using linear regression analysis while controlling for covariates (discussed below) and the first 40 genetic principal components (PCs; UKB data field 22,009) of the UKB participants to control for population stratification among White British Caucasians. We used 40 PCs given the size of the UKB sample and our concern about subtle genetic stratification even among White British Caucasians that could confound associations between PLS and various phenotypes. For parental lifespans, we have used UKB data fields as follows: “Father’s age at death” (field ID 1807) and “Mother’s age at death” (field ID 3526). Furthermore, for all the parental analysis, we have used UKB data field as follows: “Adopted as a child” (field ID 1767) to filter out non-biological parents. We only included parental lifespans on parents who had died for the regression analyses. We also tested the significance of the differences among the parental lifespan distributions between individuals in the lower and higher percentiles of PLS distributions. For the linear regression analyses, we took parental lifespan (excluding the parents who are alive) as the dependent variable and PLS as the independent variable with birth year, genotype batch, participant evaluation, and recruitment site, and the first 40 genetic PCs as covariates. We used the R package “glm” function for all regression calculations [56]. We stratified by sex in many of our analyses and also considered analyses of fathers’ and mothers’ lifespans separately. Furthermore, we performed a Cox proportional hazards (Cox-PH) survival analysis implemented in the R package “survival” [57] for the analysis of the parental lifespans using their dead/alive status and age at death or their last live recording as a censoring variable. Thus, the Cox-PH analyses take into consideration all the parents while the linear regression analysis only considered parents who are not alive.

We also examined the relationships between the PLS and disease diagnoses for conditions for which we had computed PRS values (see above) using logistic regression analysis. We focused on several common cancers (bladder, breast, colorectal, leukemia, lung, oropharyngeal, ovarian, pancreatic, prostate, testicular, and thyroid) and several common chronic non-cancerous age-related diseases (Alzheimer’s disease (Alz), atrial fibrillation (AF), coronary artery disease (CAD), heart attack, celiac disease, type 1 diabetes (t1d), and type 2 diabetes (t2d)) for which disease-specific PRS have been developed. For these analyses, we took disease-positive/negative status as the dependent variable and PLS, corresponding disease-PRS, age, sex, and first 40 PCs as independent variables. Sex was not used as a variable for the cancers which only have single-gender population in UKB, namely breast, ovarian, prostate, and testicular cancers. We constructed the PRS for the different diseases using available information derived from non-UKB data sources to avoid training bias. We also reran the analyses with simulated PLS to check the robustness of our findings. In addition, we performed a similar analysis with the same covariates to test the association between PLS and death from COVID-19. The COVID-19 data for the UKB is periodically updated, and our analyses were based on data available as of November 18, 2022.

### Simulated PLS and population structure analyses

Despite controlling for population stratification by using the genetic PCs as covariates in our various association analyses, we further investigated the PLS as possibly capturing subtle genetic ancestry information in addition to genetic effects on lifespans by testing the correlations between the 11 PLS and the first 40 genetic PCs used as covariates in our analyses. We also compared the results of these correlations with correlations between simulated PLS made by randomly choosing the same number of variants (from UKB variants) as those used in the construction of the real PLS. This provided a null distribution of correlation strengths with which we could compare the correlations with actual PLS values.

To test the correlation with parental lifespan more robustly, we created an additional type of random PLS including the real PLS and weights but only 50% of the variants being replaced by randomly selected variants. Therefore, these two categories can be considered as 100% random and 50% random PLS. We then tested the Pearson and Spearman correlation values of each of these PLS with UKB parental lifespans and iterated the process ten times. The simulated PLS data were not split between males and females but tested for associations with fathers’ and mothers’ lifespans separately.

### Individual variant association analyses

Finally, we tested each significant variant, reported in GWAS that we used to construct PLS, for association with the parental lifespans, except Timmers et al. [34] and Tesi et al. [33] as those variants are already based on these parental lifespans. We used the Plink version 2 “glm” function, taking fathers’ and mothers’ lifespans separately as the independent quantitative trait and birth year, genotyped batch, and first ten PCs as covariates and studied female and male cohorts separately.

## Results

### Correlations among PLS

The variants included in most of the PLS overlapped, but not entirely. In addition, the variants that are common among some PLS do not have equivalent weights in the scoring for the different PLS due to the different data sets used to derive each of the PLS, LD relationships between the variants, and the number and nature of the other variants considered in their construction. Table 2 (above the diagonal) contains the number of variants that overlap among the 11 PLS that we constructed in the UKB cohort. We also defined a new metric of variant weight overlap described in Methods to compare each pair of PLS in terms of the weight contribution of their shared variants. Table 2 (below the diagonal) also contains these values.

Pearson and Spearman nonparametric correlations among the PLS over all the selected UKB individuals (*N* = 408,646) are provided in Supplementary Fig. 1. As expected, all PLS pairs have positive correlations. We emphasize that some of the PLS (dl90eur, dl90eur5_8e, dl99eur, dl99eur5_8e) use overlapping sets of variants which drive these strong correlations. We note that seven PLS that use only longevity-associated variants exhibiting strong associations, namely dl90eur, dl90eur5_8e, dl99eur, dl99eur5_8e, seb, tim, and tesi (primary PLS), are strongly correlated, whereas the PLS based on genome-scale analyses using the PRS-CS algorithm exhibit lower correlations among themselves (except dl90_cs and dl99_cs) as well as with the primary PLS.

### PLS and parental lifespans

#### Correlation and simple t test analysis results suggest strong associations

Table 3 provides the sex- and parental sex-specific results of Pearson and Spearman (rank) correlations between the different PLS and parental lifespan. Although the correlation values were small, they were all positive and were all highly significant. As expected, the tim_cs PLS exhibits stronger correlation values compared to the other PLS as it consists of a large set of variants already trained on UKB parental lifespans. Tests of the difference in parental lifespan distributions between individuals in the upper and lower 10th percentiles of the distribution suggested that the mean lifespan is 0.31 to 1.98 years greater for those in the upper 10th percentile (~ 8.0 years for the overtrained tim_cs PLS; Supplementary Table 2), which is consistent with the parental lifespan being greater among individuals with higher PLS values. The *p* values of almost all the *t* tests are highly significant, ranging from < 1.0e-100 to 0.025. We note that PLS constructed from a small number of variants yield only a few different values for those PLS (e.g., for two SNP loci, there are nine different two-locus genotype combinations, so nine PLS values total in theory, though there can be more than nine due to different dosage values of alleles in the UKB genotype files). Therefore, individuals in the upper and lower 10th percentiles really reflect individuals with different PLS values. Supplementary Fig. 2 provides two graphs of these distribution differences (all the graphs are available from the authors).

**Table 3:** Pearson and Spearman correlation values between PLS and parental lifespan in the UKB (upper entry in each cell) and *p* values (lower entry in each cell)

Correlation	Pearson				Spearman			
Sex	Male		Female		Male		Female	
Parent	Mother	Father	Mother	Father	Mother	Father	Mother	Father
dl90eur	0.0123	0.0117	0.0117	0.0102	0.0138	0.0120	0.0139	0.0101
	2.709e-05	1.154e-05	1.643e-05	3.412e-05	2.477e-06	6.495e-06	3.145e-07	3.838e-05
dl90eur5_8e	0.0121	0.0118	0.0123	0.0119	0.0143	0.0118	0.0146	0.0120
	3.891e-05	9.549e-06	6.577e-06	1.316e-06	1.124e-06	9.299e-06	7.296e-08	1.082e-06
dl99eur	0.0140	0.0125	0.0115	0.0130	0.0161	0.0124	0.0145	0.0122
	1.889e-06	2.373e-06	2.462e-05	1.234e-07	4.457e-08	3.046e-06	9.728e-08	7.276e-07
dl99eur5_8e	0.0131	0.0128	0.0120	0.0132	0.0158	0.0135	0.0150	0.0133
	8.450e-06	1.406e-06	1.113e-05	7.462e-08	7.546e-08	3.618e-07	3.277e-08	6.090e-08
dl90_cs	0.0310	0.0425	0.0354	0.0388	0.0346	0.0428	0.0381	0.0397
	4.802e-26	1.008e-57	9.285e-39	4.051e-56	4.730e-32	2.426e-58	1.689e-44	9.348e-59
dl99_cs	0.0219	0.0315	0.0223	0.0279	0.0248	0.0317	0.0253	0.0297
	9.357e-14	1.701e-32	2.682e-16	9.348e-30	3.282e-17	8.402e-33	1.602e-20	1.287e-33
tim	0.0240	0.0285	0.0217	0.0317	0.0269	0.0303	0.0261	0.0316
	2.790e-16	8.924e-27	1.338e-15	5.574e-38	4.644e-20	4.476e-30	9.458e-22	7.741e-38
tim_cs	0.1760	0.1755	0.1636	0.1752	0.1953	0.1878	0.1831	0.1866
	< 1.0e-100	< 1.0e-100	< 1.0e-100	< 1.0e-100	< 1.0e-100	< 1.0e-100	< 1.0e-100	< 1.0e-100
seb	0.0111	0.0075	0.0122	0.0119	0.0128	0.0086	0.0150	0.0093
	0.00015	0.00487	7.685e-06	1.309e-06	1.337e-05	0.00116	3.199e-08	0.00017
seb_cs	0.0281	0.0333	0.0220	0.0264	0.0319	0.0335	0.0254	0.0282
	1.095e-21	6.029e-36	5.503e-16	6.410e-27	1.515e-27	2.126e-36	1.082e-20	2.088e-30
tesi	0.0196	0.0217	0.0186	0.0215	0.0231	0.0223	0.0235	0.0223
	2.265e-11	3.049e-16	7.749e-12	2.480e-18	3.358e-15	4.138e-17	5.003e-18	1.170e-19

#### Linear regression analysis reveals PLS associations

Linear regression analysis results taking parental lifespan as the dependent variable and PLS as the primary independent variable with birth year, the first 40 genetic PCs, genotype batch, and assessment center and other potential sources of confounding as covariates are provided in Table 4 for males and in Table 5 for females (Supplementary Table 1 contains the number of subjects in these analyses). UKB participants whose corresponding parent was alive at the time of the data collection were not included in these analyses. Since we included terms in the regression models for the assessment centers, genotyping batch information, and other covariates, there were too many coefficients to report for the models beyond the regression coefficients for the PLS in each model, but all the information is available as text files from the authors. All PLS exhibited weak (compared to tim_cs) yet highly significant positive associations with parental lifespans after controlling for the various potential sources of confounding (*p* values ranging from 0.0088 for the seb PLS to < 1.0e-100 for tim_cs), supporting the hypothesis that higher PLS is associated with longer parental lifespans.

**Table 4:** PLS coefficient analyses from linear regression of parental lifespans on PLS and covariates for the males in the UKB

PLS	Father’s lifespan			Mother’s lifespan		
	Estimate	Std. error	*p* value	Estimate	Std. error	*p* value
dl90eur	1.26e-01	3.43e-02	2.35e-04	1.27e-01	3.71e-02	6.05e-04
dl90eur5_8e	1.34e-01	3.43e-02	9.21e-05	1.27e-01	3.71e-02	5.88e-04
dl99eur	1.41e-01	3.43e-02	3.64e-05	1.60e-01	3.71e-02	1.72e-05
dl99eur5_8e	1.45e-01	3.42e-02	2.36e-05	1.45e-01	3.71e-02	8.90e-05
tim	3.66e-01	3.43e-02	1.23e-26	2.89e-01	3.72e-02	7.41e-15
seb	8.96e-02	4.21e-02	8.80e-03	1.30e-01	3.70e-02	4.34e-04
tesi	2.49e-01	3.42e-02	3.34e-13	2.28e-01	3.71e-02	7.99e-10
dl90_cs	4.38e-01	3.45e-02	5.94e-37	2.68e-01	3.76e-02	9.36e-13
dl99_cs	3.50e-01	3.44e-02	2.39e-24	2.05e-01	3.74e-02	4.07e-08
seb_cs	3.21e-01	3.46e-02	2.02e-20	2.25e-01	3.76e-02	2.19e-09
tim_cs	2.14	3.42e-02	< 1.00e-100	2.09	3.74e-02	< 1.00e-100

**Table 5:** PLS coefficient analyses from linear regression of parental lifespans on PLS and covariates for the females in the UKB

PLS	Father’s lifespan			Mother’s lifespan		
	Estimate	Std. error	*p* value	Estimate	Std. error	*p* value
dl90eur	9.96e-02	3.16e-02	1.61e-03	1.09e-01	3.48e-02	1.68e-03
dl90eur5_8e	1.28e-01	3.15e-02	5.15e-05	1.17e-01	3.47e-02	7.91e-04
dl99eur	1.46e-01	3.15e-02	3.67e-06	1.12e-01	3.48e-02	1.31e-03
dl99eur5_8e	1.47e-01	3.16e-02	3.07e-06	1.14e-01	3.48e-02	1.04e-03
tim	3.97e-01	3.15e-02	2.53e-36	2.53e-01	3.48e-02	3.16e-13
seb	1.37e-01	3.15e-02	1.39e-05	1.28e-01	3.48e-02	2.29e-04
tesi	2.47e-01	3.16e-02	4.78e-15	2.05e-01	3.49e-02	4.37e-09
dl90_cs	4.00e-01	3.17e-02	1.89e-36	3.47e-01	3.51e-02	5.61e-23
dl99_cs	2.99e-01	3.17e-02	3.45e-21	2.21e-01	3.51e-02	3.06e-10
seb_cs	2.23e-01	3.19e-02	2.66e-12	1.66e-01	3.53e-02	2.60e-06
tim_cs	2.12	3.14e-02	< 1.00e-100	1.96	3.50e-02	< 1.00e-100

#### Parental survival analyses reveal highly significant associations

**Fig. 1:**
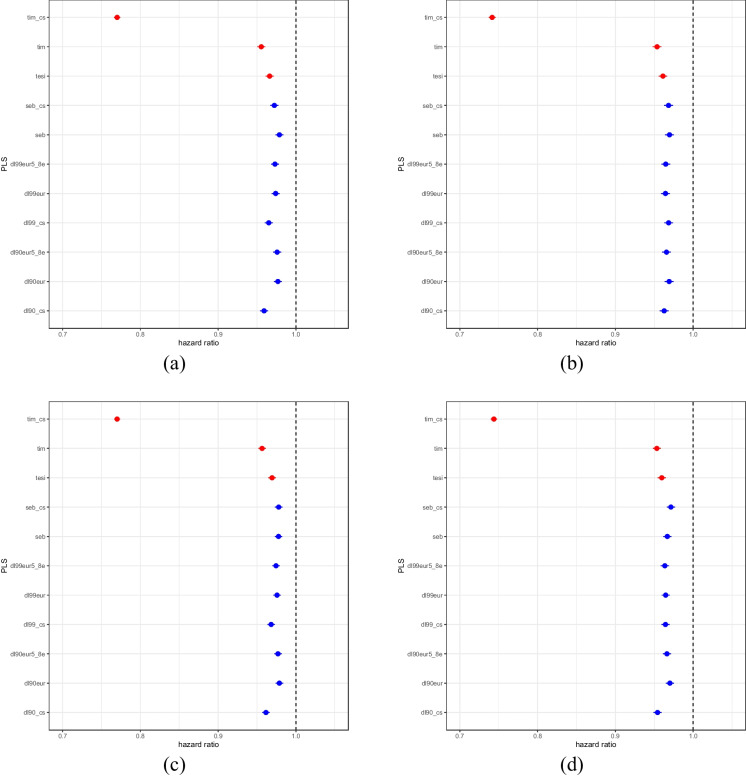
Cox-PH survival analysis hazard ratios for PLS. Dots represent the hazard ratio value with whiskers reflecting the 95% confidence intervals. **a** Fathers’ lifespan of males. **b** Mothers’ lifespan of males. **c** Fathers’ lifespan of females. **d** Mothers’ lifespan of females. The vertical line reflects a hazard ratio of 1.0. The PLS having possible training bias are denoted in red

Cox proportional hazards models for this analysis to account for right censoring of lifespans for parents who were still alive at the time of the data collection also identified very strong correlations between the PLS and parental survival (Fig. 1; Supplementary Table 1 contains the number of subjects). These analyses also considered the same covariates as the linear regression analyses. As expected, PLS have lower hazard ratios (< 1) for parental death, all with highly significant *p* values < 1e-15. Note we have used red symbols for the PLS trained on the UKB data in Fig. 1 since they suffer from overtraining but are good to contrast with the independently derived PLS. Supplementary Table 3 contains the actual HR values with confidence intervals and *p* values.

#### Individual variant association analyses

We further tested the association of each of the variants used in the PLS derived independently of UKB (dl90eur, dl90eur5_8e, dl99eur, dl99eur5_8e, seb) with UKB parental lifespans. We found that only some of variants reported in these studies exhibit independent genome-wide significant associations with UKB parental lifespans. The results are available in Supplementary Excel files for fathers-sons, fathers-daughters, mothers-sons, mothers-daughters, father with both sons and daughters, and mothers with both sons and daughters.

### PLS and disease diagnosis

#### The absence of many specific chronic diseases is associated with elevated PLS

**Fig. 2:**
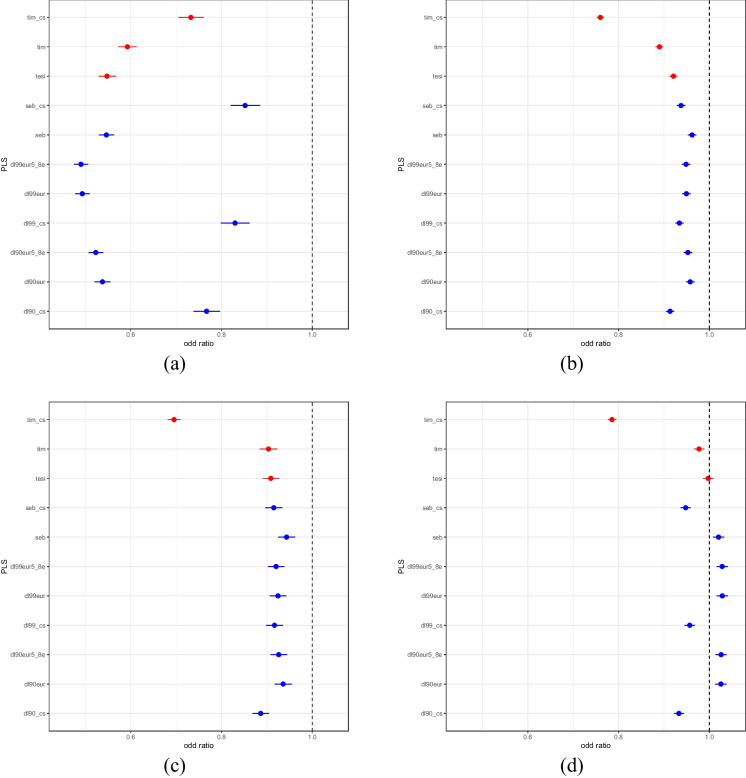
Odds ratios for the PLS based on logistic regression with **a** Alzheimer’s disease (cases = 2741, controls = 405,563), **b** coronary artery disease (CAD) (cases = 48,929, controls = 359,375), **c** heart attack (cases = 9804, controls = 397,837), and **d** type 2 diabetes (cases = 30,806, controls = 376,485). Whiskers reflect the 95% confidence intervals. The PLS having possible training bias are denoted in red

Logistic regression analyses exploring the associations between the PLS and several disease diagnoses in the UKB identified a number of strong associations but not for every disease we considered (Fig. 2). Among non-cancerous diseases, Alzheimer’s disease showed highly significant, weak negative association with all the PLS (all with *p* values < 1.74e-16), as did CAD (*p* < 3.11e-15) and heart attack (*p* < 8.06e-9). For Alzheimer’s disease analyses, we note that a few PLS include variants in LD with APOE4 variants (i.e., rs429358 and rs7412) that are known to be associated with Alzheimer’s disease. However, since we included in the logistic regression analyses Alzheimer’s PRS, which also include variants whose weights are trained for Alzheimer’s susceptibility but not longevity, we accounted for this effect. Thus, the PLS appear to have a protective effect on Alzheimer’s over-and-above PRS susceptibility effects. Although atrial fibrillation has negative associations with all PLS, some *p* values are not statistically significant, especially not after adjustment for multiple comparisons. Celiac disease and type 1 diabetes did not show clear significant negative associations with PLS while type 2 diabetes exhibited some significant positive and negative associations (data not shown for all the diseases in Fig. 2). This could be attributable to frequency of these diseases among UKB participants. The analyses using the simulated PLS showed no associations with the diseases, suggesting that our analyses with the actual PLS are robust (data not shown). PLS did not show significant associations with any of the cancer diagnoses, with the exception of the tim_cs PLS (data not shown). Graphs for all the diseases with real and simulated PLS results, as well corresponding *p* values, etc., from the logistic regression analyses are available from the authors.

#### PLS and COVID-19 deaths

**Fig. 3:**
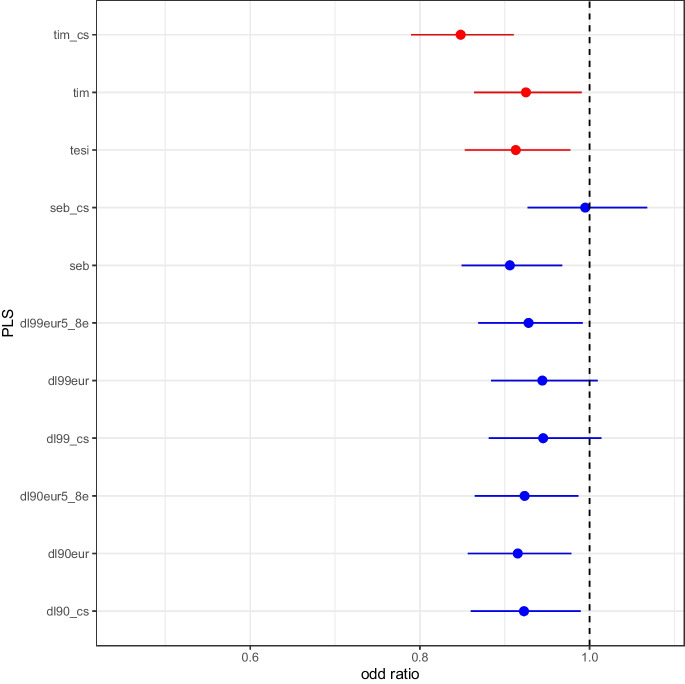
Odds ratios for the PLS based on logistic regression for COVID-19-related deaths (*N* = 1008) from COVID-19-infected people (*N* = 11,618). Whiskers reflect the 95% confidence intervals. The PLS having possible training bias are denoted in red

Logistic regression analysis with death due to COVID-19 (UKB ICD codes U071 and U072) as the dependent variable, with individuals who reported being positive for COVID-19 at least once as controls, with PLS, sex, birth year, and first 40 genetic PCs, suggested a negative correlation between PLS and death due to COVID-19. Figure 3 depicts the odd ratios and 95% confidence intervals for each of the PLS. All the odds ratios are less than 1.0, indicating a PLS association with surviving COVID, with corresponding *p* values for the PLS regression coefficients being as follows: dl90eur, 0.0091; dl90eur5_8e, 0.0177; dl99eur, 0.0908; dl99eur5_8e, 0.0266; dl90_cs, 0.0243; dl99_cs, 0.1160; tesi, 0.0086; tim, 0.0254; tim_cs; 5.667e-06; seb, 0.0031; and seb_cs, 0.8846. Thus, most of the PLS have significant *p* values, which suggests a trend towards protection against COVID-19 deaths for individuals with higher PLS.

### Simulated PLS and population structures

Our comparisons of correlations between 11 simulated PLS (using the same weights as the actual PLS but with randomly selected variants of the same number as in each actual PLS (100% random PLS)) and the 40 PCs to correlations involving the actual PLS revealed that many of the actual PLS did not exhibit associations with the PCs. However, PLS based on genome-wide SNP profiles (dl90_cs, dl99_cs, tim_cs, seb_cs) did exhibit stronger associations with PCs 4 and 5 than the simulated PLS. PCs 4 and 5 appear to capture variation among the UKB British-Caucasian sub-cohort. Supplementary Fig. 3 provides an example summary of the correlations between the first 40 PCs and dl90eur5_8e and dl90_cs for mothers’ lifespans for female subjects in the UKB. All the figures for all the PLS and for all four gender categories are available from the authors. These observations suggest that some very subtle population stratification may influence PLS constructed with many variants (e.g., dl90_cs). However, since we controlled for the PCs (i.e., including PCs 4 and 5) in our analyses, the associations between the PLS and parental longevity and disease are robust to the potential confounding effects of ancestry.

### Simulated PLS and association strength

Supplementary Table 4 and Supplementary Table 5 contain the average Pearson correlation values (with their *p* values) for the simulated PLS (100% and 50% random) settings exploring associations with fathers’ and mothers’ lifespans (note that the Spearman correlation values, which were similar, are available from the authors). Supplementary Table 4 and 5 also include the corresponding values with real PLS for comparison purposes. We only pursued these simulations with the primary PLS. As expected, 100% random PLS showed no significant correlations, having both positive and negative small values in contrast to real PLS which were comparably large, positive, and significant. Also as expected, the 50% random PLS exhibited values between the 100% random and real PLS values. These results are consistent with the real PLS exhibiting associations that are highly unlikely to be attributable to chance.

## Discussion

There is great interest in identifying genetic factors that may contribute to longevity by protecting individuals from age-related diseases or slowing their rate of aging in some way [58, 59]. Identifying genes that are protective against disease and enhance longevity in the process is difficult for many reasons. First, the complexities and expense in following cohorts of individuals and making measurements on them until they die is prohibitive in many instances. Second, the genetic bases of longevity and age-related diseases are polygenic and exhibit many overt and subtle gene × environment interactions [59, 60], which could confound the detection of any one gene. We identified variants found to be associated with longevity from GWAS and meta-analyses pursued by Deelen et al. [29], Timmers et al. [34], Sebastiani et al. [32], and Tesi et al. [33] and created PLS. These PLS capture the combined influence of the variants on the probability that an individual’s parents are long-lived and that an individual is free of life-compromising conditions. PLS are analogous to PRS which capture the combined effects of variants associated with disease and provide a summary of an individual’s genetic susceptibility to a disease [5, 6, 15, 61].

We tested the associations between 11 different PLS constructed from the different longevity GWAS, as well as ways of identifying variants to be included in a PLS, and parental lifespan and different diseases in the UKB. We acknowledge that 3 of these 11 PLS (tim, tim_cs, and tesi) were based on UKB parental lifespan data and hence suffer from overfitting. We find strong evidence that all the PLS are associated with parental lifespan in the UKB, including those that were trained on independent data sets and not the UKB data. However, the associations between the PLS and longer parental lifespan are very small in terms of the additional years of life they are, on average, associated with (~1 year). In addition, the effects of the various PLS are similar, but their compositions are very different in terms of the SNPs used to construct them. This suggests that the construction of more reliable PLS may require larger data sets to capture the bulk of genetic variants that affect longevity.

Importantly, the associations of the PLS with diseases are independent of actual disease risk based on PRS, since we included both PLS and disease-specific PRS in our analyses. In addition, these PLS are also negatively associated with different disease diagnoses in the UKB, including Alzheimer’s disease, CAD, heart attack, and death from COVID-19, but not with cancers. COVID-19 deaths have been reported to be more frequent among older individuals with comorbidities and underlying issues [62]; however, genetic factors and health-compromising issues in younger individuals can also contribute to COVID-19-related deaths [63]. We also note that there are many factors that contribute to infectious disease susceptibility and severity of disease that were not accounted for in our analyses that could distinguish those who died from COVID-19 and those who did not (e.g., vaccinations, co-infections, comorbid conditions, and different variants of the virus). Despite this, our findings raise important questions about the functional basis of the variants contributing to longevity (i.e., those used to form the different PLS) and their protective effect on the development of specific diseases, but not all diseases. The lack of association between PLS and cancer diagnosis could be attributable to insufficient power and/or to the myriad environmental, lifestyle, and/or behavioral factors that contribute to cancer, as well as a stochastic or purely “random” component to cancer initiation and development, but clearly more work needs to be pursued to address this [64–67].

Our findings are consistent with other studies focusing on genetic and non-genetic factors contributing lifespan that have used different data sets, different sets of PRS, and alternative strategies for constructing PLS. For example, one study using data on a 5-year follow-up within the UKB considered models for predicting death during that follow-up period and found that many different factors, not including genetics, were predictive of death [68]. This suggests that more sophisticated and genetically informed models have the potential to add insights to factors contributing to lifespan since many of the traits that were predictive of mortality in this study are known to have genetic determinants (e.g., blood pressure and hypertension) [68]. The studies by Timmers et al. [34, 69] focusing on genetic factors influencing parental lifespan in the UKB that we, in part, leverage in the present analyses are evidence for this. A recent study of two independent cohorts in Australia, the Sydney Centenarian Study and the Sydney Memory and Ageing Study, found evidence for association between polygenic background and exceptional longevity (EL) but did not find evidence that individuals exhibiting EL had significantly less risk of disease based on disease-specific PRS [42]. A follow-up study by the same group found that the PLS they derived previously was associated with a favorable metabolic profile [31]. Another recent independent study in Croatia found evidence that long-lived individuals (90–95 years or older) harbored a unique genetic profile [70], and yet another recent study involving a German cohort also found evidence for an association between a PLS they derived and longevity [37].

Unlike the studies in Australia, many other studies have found evidence that long-lived individuals possess lower disease-specific PRS [71–73]. In addition, a study involving a large cohort of twins found that non-genetic factors, including routine blood-based clinical chemistries such as C-reactive protein, gamma-glutamyl transferase, glucose, and alkaline phosphatase, were more predictive of lifespan and longevity than PLS that they derived, although many clinical chemistries are, in fact, known to have genetic determinants [74]. We find that the relationships between PLS, disease-specific PRS, and longevity are complex, and that elevated PLS may mitigate the effects of elevated PRS (Fig. 2). However, we believe more research is necessary to sort of the even more complex interplay between PLS, PRS, non-genetic factors, labile disease biomarkers such as cholesterol level and CRP, and longevity.

We acknowledge that our association analyses involving PLS trained on UKB parental lifespan data (tim, tim_cs, and tesi) suffer from training bias when testing them for association with parental lifespan and diseases in the UKB. In addition, we also recognize that the association studies involving the PLS trained on the UKB with disease diagnosis may suffer from survivor bias [75, 76]. However, the fact that different PLS trained on data sets independent of the UKB and the fact that these PLS did not all include a common set of variants and weights suggest that PLS that are associated with lifespan and protection from disease in a robust way and not simply attributable to survivor bias can be constructed. Some SNP effects, however, may reflect LD to functional SNPs common to different PLS. In addition, although we found evidence that some of the PLS were associated with the genetic backgrounds of individuals in the UKB, this effect was minor, and we controlled for these associations in our analyses exploring PLS parental lifespans and disease diagnoses by using PCs capturing subtle ancestral differences in the UKB.

Using parental lifespan as a proxy for individual lifespan is a limitation of the study, but it can be argued that our results are therefore conservative as much greater effects would have likely been observed if lifespan and genotype data are from the same individuals. When examining the lifespan of parents, deaths due to non-natural causes may distort the results, but we believe that when considering a large cohort, such cases are proportionally small and their effects are minor. It should also be kept in mind that deaths due to non-natural causes, such as accidents or violence that are not acknowledged in the UKB, undermine the use of reported age at death for longevity studies if not censored or accommodated in relevant analyses. However, the effect of such a phenomenon would be to create noise in the lifespan data and thereby reduce power to detect positive associations between PLS and parental lifespan, as well as negative associations between PLS and diseases. The fact that we have identified very strong and consistent associations suggests that this phenomenon is not so pronounced to completely reduce the power to detect associations. Finally, we acknowledge that since we did not use all of the variants associated with the various PLS obtained from the longevity GWAS sources for analyses of the UKB genotype data, there may be some bias in our analyses. However, the optimal way of choosing replacement variants based on, e.g., LD relationships and recomputing effect sizes (i.e., weights) was not the focus of this paper. In addition, by excluding variants in the PLS calculations, we believe that our association analyses are conservative, such that more complete PLS would likely show stronger correlations, and hence our analyses are more likely to suffer from false negative rather than false positive bias. Thus, the associations we found are not likely to be due to survivor bias or false positives attributable to SNP genotype availability and population stratification in the UKB.

There are many follow-up studies that make sense to pursue in the wake of our findings. For example, functional evaluation of the variants used in the PLS could be pursued, although the penetrance of any one variant used in a PLS may be slight. The PLS need to be both constructed and explored in non-European populations as well as in the context of any potential gene × environment interactions the variants might exhibit individually or collectively. In addition, better and more sophisticated ways of constructing PLS should be pursued, including those that aggregate information from different data sets. While one could explore genetic correlations between longevity-associated phenotypes and disease traits to obtain better insight into the relationship between genes and the protective effects of variants in those genes [60], it has recently been shown that genetic correlation analysis can be problematic since genetic correlations can be confounded by assortative mating [77]. Our studies included PLS derived from centenarians and long-lived individuals generally, but they could be studied not only with younger cohorts of individuals to see if they impact the health trajectories of younger individuals in different contexts, but also as part of clinical epidemiology studies making use of PRS to see if the PLS can contextualize or risk stratify individuals based on their PRS.

### Supplementary Information

Below is the link to the electronic supplementary material.

Supplementary file1 (DOCX 155 KB)

Supplementary file2 (XLSX 16 KB)

Supplementary file3 (XLSX 15 KB)

Supplementary file4 (XLSX 15 KB)

Supplementary file5 (XLSX 15 KB)

Supplementary file6 (XLSX 17 KB)

## Data Availability

The summary statistic data we used to derive the various PLS are discussed in the publications referenced for each PLS. Data from the UK Biobank must be accessed through the UK Biobank permissioning process. Additional information about the derivation of the PLS not in the paper or supplementary material can be obtained from the authors.
