# Supplementary material for: The relationship between 11 different polygenic longevity scores, parental lifespan, and disease diagnosis in the UK Biobank
Source: GeroScience. 2024 Mar 7;46(4):3911–27. doi: 10.1007/s11357-024-01107-1 (PMC11226417; doi:10.1007/s11357-024-01107-1)
Supplement: Supplementary file 1 — Supplementary file1 (DOCX 155 KB) [file 11357_2024_1107_MOESM1_ESM.docx]

**SUPPLEMENTARY TABLES AND FIGURES**

**Supplementary Table 1** Number of subjects used in parental lifespan analysis. Cox-PH model have more subjects as it additionally contains subjects having alive parents

| **Analysis** | **Fathers’ lifespan of**  **males** | **Mothers’ lifespan of**  **males** | **Fathers’ lifespan of females** | **Mothers’ lifespan of females** |
| --- | --- | --- | --- | --- |
| All analysis having parental lifespans | 141545 | 115936 | 165321 | 135140 |
| Cox-PH  Survival Analysis | 178183 | 180127 | 211242 | 215841 |

**Supplementary Table 2** Difference between the mean lifespan (in years) and corresponding p-values (within parentheses) for individuals in the upper and lower 10^th^ percentile of the distribution of each PLS

| **PLS** | **Fathers’ lifespan of males** | **Mothers’ lifespan of males** | **Fathers’ lifespan of females** | **Mothers’ lifespan of females** |
| --- | --- | --- | --- | --- |
| dl90eur | 0.5975  (5.721e-05) | 0.6768  (3.623e-05) | 0.5448  (6.700e-05) | 0.5753  (0.000174) |
| dl90eur5_8e | 0.5059  (0.000533) | 0.5223  (0.001157) | 0.4794  (0.000395) | 0.3704  (0.011055) |
| dl99eur | 0.5434  (0.000226) | 0.4918  (0.001939) | 0.5756  (2.746e-05) | 0.436  (0.003413) |
| dl99eur5_8e | 0.559  (0.000147) | 0.5663  (0.000449) | 0.5554  (5.057e-05) | 0.3143  (0.024795) |
| tim | 1.0882  (1.350e-12) | 1.2701  (6.665e-14) | 1.5494  (9.942e-28) | 0.9939  (3.715e-10) |
| seb | 0.3193  (0.020119) | 0.3948  (0.010890) | 0.5186  (0.000138) | 0.474  (0.001559) |
| tesi | 0.9758  (1.681e-10) | 0.8404  (4.123e-07) | 0.8986  (1.465e-10) | 0.8334  (1.014e-07) |
| dl90_cs | 1.9756  (5.230e-38) | 1.4469  (1.890e-17) | 1.7259  (7.943e-34) | 1.6993  (3.172e-26) |
| dl99_cs | 1.5937  (2.406e-25) | 0.8151  (1.1455e-06) | 1.0757  (2.252e-14) | 1.0979  (4.741e-12) |
| seb_cs | 1.5525  (1.430e-23) | 1.1483  (1.296e-11) | 1.2044  (1.754e-17) | 1.0946  (3.942e-12) |
| tim_cs | 8.1192  (<1.0e-100) | 7.989  (<1.0e-100) | 8.0618  (<1.0e-100) | 7.7624  (<1.0e-100) |

**Supplementary Table 3** Hazard ratios, 95% confidence intervals (in parentheses) and p-values (second line) for the PLS in a Cox-PH model for parental lifespans for each sex and parental sex. The models included covariates (not shown)

| **PLS** | **Fathers’ lifespan of males** | **Mothers’ lifespan of males** | **Fathers’ lifespan of females** | **Mothers’ lifespan of females** |
| --- | --- | --- | --- | --- |
| dl90eur | 0.9769(0.9718 - 0.9820)  1.456948e-18 | 0.9691(0.9636 - 0.9747)  1.390097e-26 | 0.9787(0.9740 - 0.9835)  2.711942e-18 | 0.9701(0.9650 - 0.9753)  1.178452e-28 |
| dl90eur5_8e | 0.9759(0.9708 - 0.9810)  4.359546e-20 | 0.9657(0.9602 - 0.9713)  9.330730e-33 | 0.9770(0.9723 - 0.9817)  2.909761e-21 | 0.9663(0.9612 - 0.9715)  2.130227e-36 |
| dl99eur | 0.9740(0.9690 - 0.9791)  3.675637e-23 | 0.9644(0.9589 - 0.9700)  4.743688e-35 | 0.9758(0.9711 - 0.9805)  2.613422e-23 | 0.9647(0.9596 - 0.9699)  1.549913e-39 |
| dl99eur5_8e | 0.9731(0.9681 - 0.9782)  1.070715e-24 | 0.9648(0.9592 - 0.9703)  1.908656e-34 | 0.9744(0.9698 - 0.9791)  6.235173e-26 | 0.9636(0.9585 - 0.9688)  5.500766e-42 |
| tim | 0.9555(0.9506 - 0.9605)  1.120302e-65 | 0.9536 (0.9481 - 0.9591)  8.804679e-59 | 0.9565 (0.9519 - 0.9612)  7.204371e-73 | 0.9533 (0.9483 - 0.9584)  4.041044e-69 |
| seb | 0.9788 (0.9738 - 0.9839)  7.757237e-16 | 0.9695 (0.9640 - 0.9751)  4.527804e-26 | 0.9777 (0.9730 - 0.9824)  4.594391e-20 | 0.9668 (0.9617 - 0.9720)  2.388296e-35 |
| tesi | 0.9663 (0.9613 - 0.9714)  4.903692e-38 | 0.9611 (0.9556 - 0.9667)  1.237554e-41 | 0.9694 (0.9647 - 0.9741)  1.294187e-36 | 0.9597 (0.9546 - 0.9648)  2.238742e-51 |
| dl90_cs | 0.9591 (0.9541 - 0.9642)  1.074259e-54 | 0.9627 (0.9572 - 0.9683)  1.255189e-37 | 0.9615 (0.9568 - 0.9662)  1.118818e-56 | 0.9542 (0.9491 - 0.9594)  1.066795e-65 |
| dl99_cs | 0.9651 (0.9601 - 0.9702)  1.420577e-40 | 0.9684 (0.9628 - 0.9740)  1.117764e-27 | 0.9681 (0.9635 - 0.9728)  3.075547e-39 | 0.9644 (0.9593 - 0.9696)  5.212426e-40 |
| seb_cs | 0.9723 (0.9672 - 0.9774)  1.015624e-25 | 0.9683 (0.9627 - 0.9740)  1.932336e-27 | 0.9778 (0.9731 - 0.9826)  2.231667e-19 | 0.9714 (0.9661 - 0.9766)  5.925127e-26 |
| tim_cs | 0.7702 (0.7661 - 0.7742)  <1.0e-100 | 0.7416 (0.7373 - 0.7459)  <1.0e-100 | 0.7701 (0.7664 - 0.7739)  <1.0e-100 | 0.7437 (0.7397 - 0.7478)  <1.0e-100 |

**Supplementary Table 4** Pearson Correlation between 100% random, 50% random, and real PLS with UKB fathers’ lifespans. The cohort has not divided to males and females. Each correlation value is in the first line and corresponding p-value within brackets is in the second line

| PLS | 100% Random | 50% Random | Real |
| --- | --- | --- | --- |
| dl90eur | 0.0008  (0.349916) | 0.0065  (0.070077) | 0.0109  (1.37e-09) |
| dl90eur5_8e | -0.0003  (0.462916) | 0.0096  (0.000674) | 0.0119  (4.78e-11) |
| dl99eur | -0.0005  (0.405328) | 0.0093  (0.000487) | 0.0128  (1.31e-12) |
| dl99eur5_8e | 0.0001  (0.255489) | 0.0088  (0.004318) | 0.0130  (5.02e-13) |
| seb | 0.0001  (0.540441) | 0.0067  (0.007642) | 0.0099  (4.58e-08) |
| tesi | -0.0001  (0.437851) | 0.0126  (0.021499) | 0.0216  (4.07e-33) |
| tim | -0.0008  (0.365912) | 0.0192  (3.66e-12) | 0.0301  (1.19e-62) |

**Supplementary Table 5** Pearson Correlation between 100% random, 50% random, and real PLS with UKB mothers’ lifespans. The cohort has not divided to males and females. Each correlation value is in the first line and corresponding p-value within brackets is in the second line

| PLS | 100% Random | 50% Random | Real |
| --- | --- | --- | --- |
| dl90eur | 0.0006  (0.431802) | 0.0067  (0.126314) | 0.0119  (2.23e-09) |
| dl90eur5_8e | -0.0002  (0.424356) | 0.0100  (0.006806) | 0.0121  (1.48e-09) |
| dl99eur | -0.0000  (0.546012) | 0.0089  (0.007281) | 0.0126  (3.13e-10) |
| dl99eur5_8e | 0.0008  (0.522517) | 0.0076  (0.062363) | 0.0124  (5.48e-10) |
| seb | -0.0013  (0.448076) | 0.0083  (0.025703) | 0.0116  (6.03e-09) |
| tesi | 0.0000  (0.382212) | 0.0112  (0.054735) | 0.0190  (1.78e-21) |
| tim | -0.0005  (0.417789) | 0.0146  (3.46e-05) | 0.0227  (6.05e-30) |

**Supplementary Fig 1** Pearson (above the diagonal) and Spearman (below the diagonal) correlations among PLS within the UKB


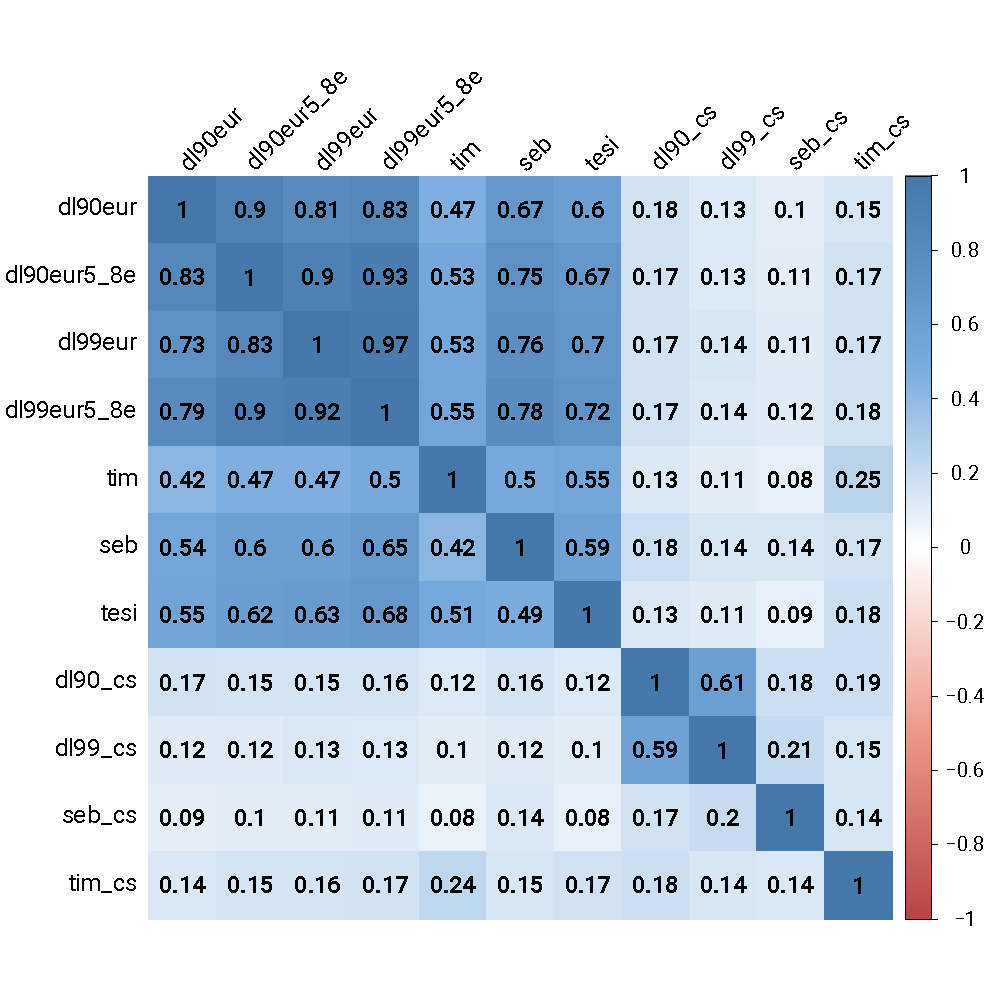


**Supplementary Fig 2** Fathers’ Lifespan distribution difference between lowest 10^th^ percentile and highest 10^th^ percentile of PLS of their sons: (a) seb_cs, (b) tim_cs. Supplementary Table 2 contains all the values for all the PLS

(a) seb_cs (b) tim_cs

**Supplementary Fig 3** Correlation strengths (positive side: Real PLS, negative side: Simulated PLS) between first 40 PCs with (a) dl90eur5_8e and (b) dl90_cs for mothers’ lifespans of females in UKB. Black vertical lines show strengths of correlation between corresponding real PLS and corresponding parents’ lifespans

1. dl90eur5_8e (b) dl90_cs
